# The aging human body shape

**DOI:** 10.1038/s41514-020-0043-9

**Published:** 2020-03-24

**Authors:** Alexander Frenzel, Hans Binder, Nadja Walter, Kerstin Wirkner, Markus Loeffler, Henry Loeffler-Wirth

**Affiliations:** 1 Interdisciplinary Centre for Bioinformatics, Leipzig University, Härtelstraße 16–18, 04107 Leipzig, Germany; 2 LIFE, Leipzig Research Center for Civilization Diseases, Leipzig University, Philipp-Rosenthal-Straße 27, 04103 Leipzig, Germany; 3 Faculty of Sport Science, Leipzig University, Jahnallee 59, 04109 Leipzig, Germany; 4 Institute for Medical Informatics, Statistics and Epidemiology, Leipzig University, Härtelstraße 16–18, 04107 Leipzig, Germany

**Keywords:** Health care, Ageing

## Abstract

Body shape and composition are heterogeneous among humans with possible impact for health. Anthropometric methods and data are needed to better describe the diversity of the human body in human populations, its age dependence, and associations with health risk. We applied whole-body laser scanning to a cohort of 8499 women and men of age 40–80 years within the frame of the LIFE (Leipzig Research Center for Civilization Diseases) study aimed at discovering health risk in a middle European urban population. Body scanning delivers multidimensional anthropometric data, which were further processed by machine learning to stratify the participants into body types. We here applied this body typing concept to describe the diversity of body shapes in an aging population and its association with physical activity and selected health and lifestyle factors. We find that aging results in similar reshaping of female and male bodies despite the large diversity of body types observed in the study. Slim body shapes remain slim and partly tend to become even more lean and fragile, while obese body shapes remain obese. Female body shapes change more strongly than male ones. The incidence of the different body types changes with characteristic Life Course trajectories. Physical activity is inversely related to the body mass index and decreases with age, while self-reported incidence for myocardial infarction shows overall the inverse trend. We discuss health risks factors in the context of body shape and its relation to obesity. Body typing opens options for personalized anthropometry to better estimate health risk in epidemiological research and future clinical applications.

## Introduction

Human body dimensions and shape vary between individuals in an age-dependent manner. Body size and shape are governed by genetic and environmental factors, including lifestyle with potential impact for health. There is growing evidence that body shape and regional body composition are strong indicators of metabolic health^1,2^. For example, overweight and obesity increase risks for developing metabolic and cardiovascular diseases in an age-dependent manner^3^. Simple anthropometric measures such as the body mass index (BMI) and waist circumference are often used to define the obesity status of a person. However, it turned out that about 10% of BMI-defined obese individuals of European ethnicity are healthy in terms of their metabolic state, while another nearly 10% have a normal BMI but are metabolically unhealthy^4,5^. Health risk obviously associates in a more complex way with human body dimensions and depends, for example, on the relation between fat and muscles and their distributions along the body^4^. For example, upper body and lower body fat depots show opposite associations with risk for diabetes and cardiovascular diseases^6–8^.

Fat distribution can be analyzed in detail using imaging techniques, such as computed tomography and magnetic resonance imaging, which are relatively expensive methods requiring expert skills and which are therefore difficult for application in large population studies. Other methods, such as dual-energy X-ray absorptiometry (DXA) and bioelectrical impedance analysis, represent interesting options of estimating “internal” tissue distribution in the human body. Three-dimensional (3D) whole-body laser scanning provides another promising technique for evaluating “external” body shape by granting the opportunity to assess dozens of anthropological body measures at once with high accuracy and within only a few seconds of time^9^. Body scanning is utilized in medical application, for example, for cosmetic and reconstructive surgery^10,11^, and increasingly in health research to study anthropometry of hundreds to thousands of participants in epidemiological cohort studies^9,12^ to assess their possible relevance for health risk prediction.

The capability of 3D laser scanning anthropometry arises from the vast number of measured body surface dimensions that allow discovery of health risk phenotypes beyond simple, one-dimensional classification schemes based on the waist-to-hip ratio (WTH) or the BMI. However, current applications usually do not consider the whole information provided by the set of body measures and instead use only a small part of them often to extract only body indices such as BMI or WTH^13–16^. 3D body scans were applied in the Leipzig Research Center for Civilization Diseases (LIFE), conducting the largest population-based study with extensive phenotyping of urban individuals in Germany^17^.

Previously, we proposed a concept of human body types based on a machine learning to extract meta-measures from the LIFE-ADULT body scanning data^18^. However, it remains unclear how body shapes change upon aging, and how body types describe the aging process.

Health scientists and epidemiologists increasingly use a Life Course approach to interpret anthropometrical data because body measures such as weight, height, and BMI in earlier stages of life seem to affect diseases later in life, such as obesity, type 2 diabetes, hypertension, or stroke^19^. Developmental trajectory types enabled to assess the relation between body shape and the mortality risk^20^. The results indicate the health benefit of body shape management across the lifespan, but they also underline the necessity of developing elaborated measures of body shapes and their age-depending characterization.

In this publication, we aim at studying and characterizing systematic alterations of anthropometric measures as a function of participant’s age using cross-sectional data derived from 3D laser scanning technique of the LIFE-ADULT cohort. Hereby, we direct our special focus towards alterations of body shapes of participants based on their stratification into body types according to our previous classification scheme.

## Results

### Aging as seen by body indices

**Fig. 1:**
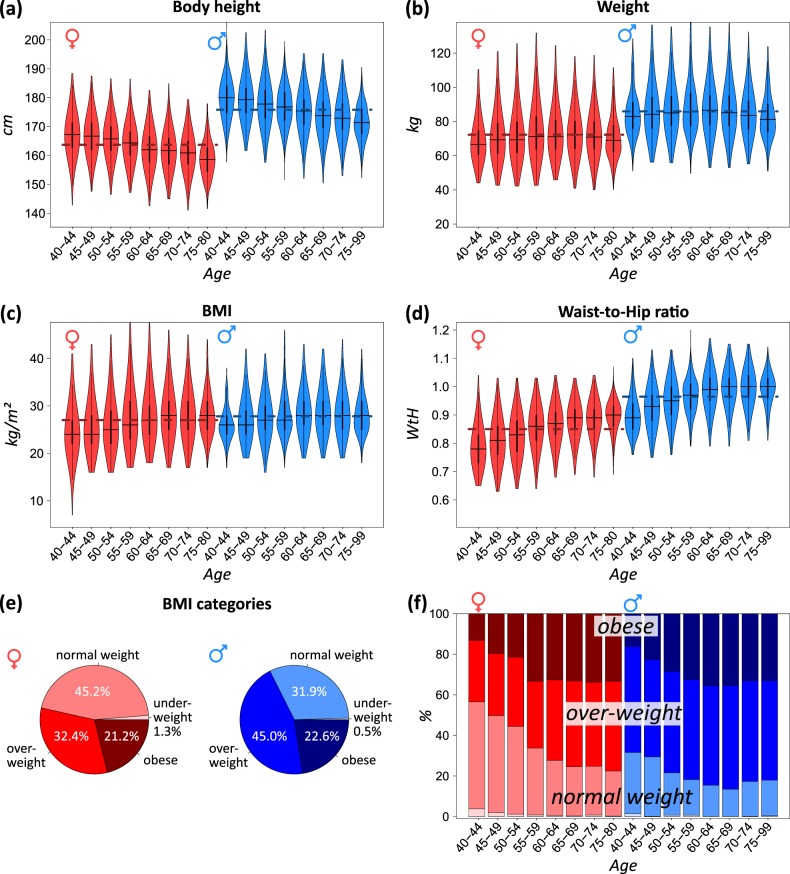
Classical anthropometric measures as a function of age. **a** Body height, **b** weight, **c** BMI, and **d** waist-to-hip circumference ratio are given as violin plots stratified by sex and age, respectively. Dashed horizontal lines refer to mean values for women and men, respectively. **e** The overall distribution of participants classified as underweight (BMI <18.5), normal weight (18.5 ≤ BMI < 25), overweight (25 ≤ BMI < 30), and obese (BMI ≥30) according to WHO classification^53^. **f** BMI categories stratified by age.

The LIFE-ADULT cohort included 10,000 participants sampled from the population of Leipzig. This cross-sectional study covers an age range from mid-age (about 40 years) up to elderly (80 years) men and women. Basic characteristics of the sampling are summarized in Table 1. 3D body scanning data is available for 8499 participants, which were stratified by sex and age to estimate the alterations of “classical” anthropometrical characteristics (body height, weight, BMI, and waist-to-hip circumference ratio (WTH)) upon aging (Fig. 1). Body heights of both sexes start to decrease from an age of about 50 years. The lower quantile of body height in younger participants (<50 years) approximately corresponds to the upper quantile in older ones (>70 years). The weight of the participants alters in an opposed fashion. It increases with age up to 55–59 years, and then it declines. Combination of height and weight results in increasing BMI values up to about 60 years and virtually invariant BMI values for older people. Such a levelling off behavior of BMI at about 60 years followed by a slight decay was found also in other studies and seems to reflect rather aging-related physiological changes than changes of lifestyle (e.g., due to retirement)^21–23^.

**Table 1:** Basic characteristics of the LIFE-ADULT study population.

	Male	Female
Total study population	4766	5234
Age (years)	57 ± 13	56 ± 12
Height (cm)	176 ± 7	165 ± 7
Weight (kg)	86 ± 14	71 ± 14
Waist circumference (cm)	101 ± 12	91 ± 13
Body mass index (kg/m^2^)	28 ± 4	26 ± 5
Waist-to-hip ratio	0.96 ± 0.08	0.84 ± 0.08
Step number	9683 ± 4011	9903 ± 3682
Metabolic equivalent	1.41 ± 0.25	1.35 ± 0.24
Alcohol consumption (g/day)	18.8 ± 21.7	5.9 ± 10.0
Reported previous myocardial infarction	192 (4.0%)	59 (1.1%)
Smoker/ex-smoker	1089 (22.8%)/1715 (36.0%)	1016 (19.4%)/1090 (20.8%)

Mean values ± standard deviation.

*BMI* body mass index, *WTH* waist-to-hip ratio, *MET* metabolic equivalent.

BMI curves of both sexes are very similar; however, men show a slightly higher mean BMI than women. The age dependency of WTH resembles that of BMI, where, however, men show typically markedly higher values compared to women. Among people older than 50 years, more than 50% show an “apple-like” body shape (WTH ≥0.8 and 0.9 in women and men, respectively), which is found to associate with higher health risk^24^.

The body indices remain, on average, virtually invariant for women and men older than 60 years, which makes them unsuitable to discriminate age-dependent trends for elderly people.

Overall, about 21–23% of women and men are obese, while a markedly higher fraction of about 45% of men are overweight compared with 32% of women. The relative amount of obese people increases with age, while that of normal weight ones decreases mainly up to an age of about 60 years. More than 25% of participants older than 60 years are obese (BMI ≥30). In summary, standard body indices reflect typical alterations of body dimensions upon aging, such as decreasing body height and increasing WTH, sex specifics, and also deviations from linear changes as, for example, observed for elderly people. Especially WTH, but also BMI, show sex-specific differences with relation to obesity. In summary, we find age-related trends of decreasing body height and weight. This single-feature related anthropometrical information is however relatively rough and not sufficient for a detailed evaluation of changes of the body shape upon aging.

### Aging body shapes

**Fig. 2:**
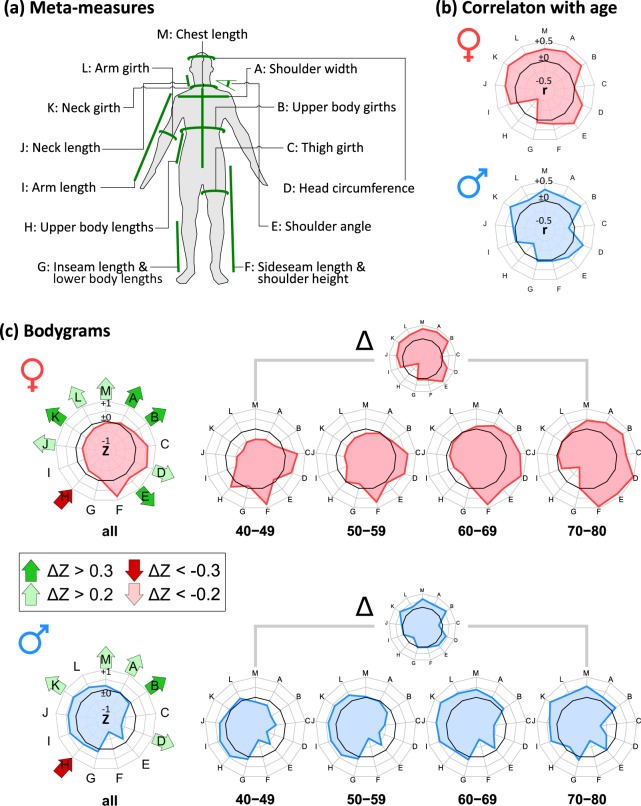
Alterations of body measures with age. **a** Assignment of the 13 meta-measures defined previously. **b** Correlation of the meta-measures with age visualized as polar diagram. The black polygon refers to *r* = 0. **c** The “bodygram” visualizes the meta-measures in *Z*-units as a polar diagram. The black polygon refers to *Z* = 0. Mean bodygrams of male and female participants averaged over all ages (left part) and after stratification into age decades (right part) reveal gender-specific differences and age-dependent changes of the meta-measures. Difference Δ-bodygrams visualize the changes of the meta-measures between the 40–49 and 70–80 years intervals. The green and red arrows in the left part highlight the most pronounced differences (Δ*Z* >±0.2).

Next, we analyzed age-related alterations of the body meta-measures, which distribute virtually over all parts of the body (Fig. 2a). Most of them positively correlate with age (Pearson’s correlation coefficients between 0.2 and 0.5, whereby correlation is stronger for women than for men in most cases, Fig. 2b). In contrast, thigh girth and upper body lengths decrease with age on the average as indicated by negative correlation coefficients.

For visualization of the meta-measures and of their changes, we use a polar diagram termed “bodygram,” where each axis refers to one meta-measure (Fig. 2c). The bodygrams reveal marked sex-specific differences such as larger dimensions of the upper body (meta-measures H–M) in men, and larger girth and length dimensions of the legs (meta-measures C and F) in women (Fig. 2c, left part), meaning that the leg measures were larger in women in relation to their body height. For an age-dependent view, we generated mean bodygrams averaged over decadal age intervals (Fig. 2c, right part), and difference bodygrams between the youngest (40–49 years) and oldest (70–80 years) strata (Fig. 2c). Interestingly, these Δ-bodygrams are very similar for women and men, indicating similar changes of the body measures upon aging despite the distinct gender specifics of the bodygrams. In correspondence with the correlation analysis, we found that all meta-measures increase upon aging, except for meta-measure H estimating upper body lengths. These opposite trends indicate reshaping of the body towards a smaller torso in relation to body size in older individuals. The increasing meta-measures collect mainly girth measures, reflecting a gain of body volume and weight as discussed above. Other meta-measures such as J and L (neck length and arm girths, respectively) markedly increase in women, while C (thigh girth) increases typically in men. The latter alterations reflect redistributions of body mass from legs towards the torso, or, in other words, the shift from a pear-like towards an apple-like body shape.

Overall, we documented alterations of the anthropometrical meta-measures extracted from 3D body scanning. They reflect marked sex-specific differences of the body shapes as expected, especially the broader upper male body and the larger dimension of female legs. At the same time, we see similar reshaping trends upon aging in both sexes, namely increasing body girths and a (relative) shortening of the upper body.

### Aging body types

**Fig. 3:**
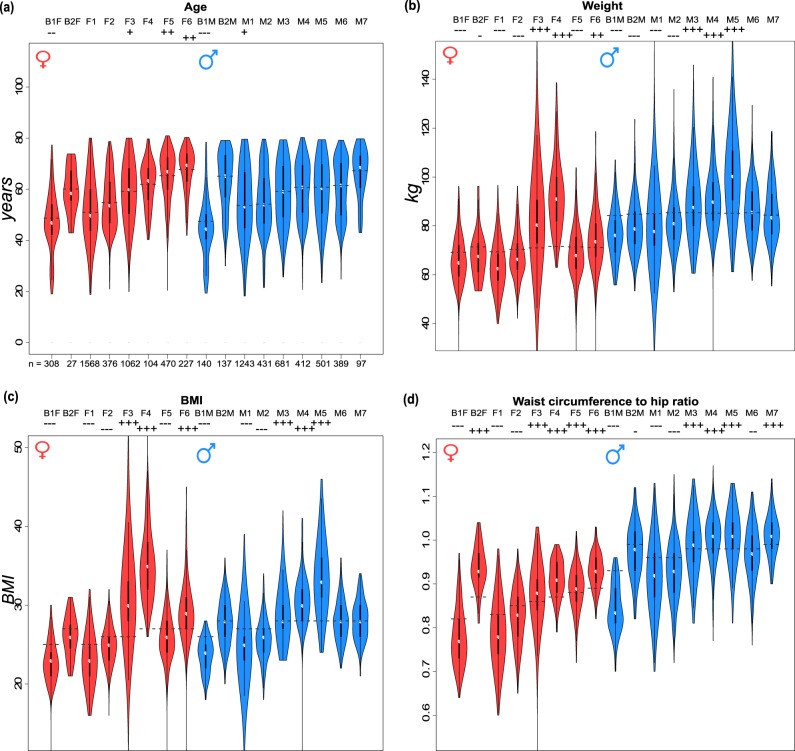
Anthropometric parameters of the body types. **a** The age dependence of F-types (F1–F6) is more pronounced than that for men (M1–M7). **b**, **c** F3 and F4 are obese types among the F-types with a high variability of F3, while M5 is the most obese M-type. **d** The WTH data do not reflect these characteristics. *P* values of differences between the body types groups and age-matched reference groups are indicated as signs in the head line of each plot (*p* < 0.1 (+/−), <0.01 (++/–), and <0.001 (+++/−−−).

In the next step, we aimed to describe age-related alterations of the 15 body types identified previously to describe the heterogeneity of body shapes observed in the population of Leipzig^18^ (see also Supplementary Fig. 1). Each of the body types collects different age strata of participants showing, however, large variances and broad mutual overlaps (Fig. 3a). We ordered the body types with increasing mean age of their members. Female body types (F-types) show a broader range of mean ages, whereas male body types (M-types) are more uniform. The gender-unspecific (B) body types collect either younger (B1) or older (B2) people and were considered separately for women (B1F, B2F) and men (B1M, B2M). Variability of M-types is higher than that of female ones, except for F3, which collects overweight and obese women of all ages. Age-related changes of BMI are small compared to variabilities of the body types (see below and Supplementary Fig. 2). Two F-types (F3 and F4) and one M-type (M5) collect mainly obese individuals (BMI >30 kg/m^2^). Interestingly, the WTH ratios do not reflect obese characteristics of these body types compared with the other, non-obese ones. Body types F5, F6, B2M, and M7 collect the highest fractions of people older than 70 years (Supplementary Fig. 1).

Detailed bodygram analysis of the different body types reveals type-specific changes upon aging, where a part of them is characterized by increasing values of the meta-measures, while others are dominated by decreasing or virtually age-invariant meta-measures (Supplementary Fig. 6). The shoulder angle, for example, increases with age, leading to more hanging shoulders for older people. Also, chest and arm lengths are growing measures reflecting the relative increase of the upper body. Decrease of the dimensions of the lower part of the body is reflected by decreasing thigh girths in men. Overall, aging body types are characterized by the shift of body proportions towards a larger upper part and smaller legs, which become relatively short and lean. Some of the meta-measures reveal gender-specific alterations, such as upper body girths, which increases typically in the M-types reflecting the shift into apple-like body shapes. Other meta-measures, for example, arm length, arm girths, neck girth, and neck length specifically change in F-typess and partly reflect the increase of the upper body’s size. In general, F-types seem to underlie stronger changes than male ones.

Table 2 summarizes the characteristic body shape changes observed. The major characteristics of the body types are virtually age independent. They maintain and partly amplify their most prominent characteristics in elderly people. For example, body types with tall and lean shape (B1, F1, and M2) become longer and/or leaner (longer chest and upper body). Moreover, men with a broad neck (M4 and M7) keep this property, and participants with a massive upper body (F3 and M5) additionally get leaner legs. Overall, these results indicate that, upon aging, slim body shapes remain slim and partly tend to become even more lean and fragile, while obese body shapes remain obese. For most of the body types, we observe sex-independent changes upon aging as described in the previous subsection. Stratification of individuals into body types provides a detailed picture of aging body shapes.

**Table 2:** List of body types and associated characteristics of body shape.

Body type	General characteristics and age-related change	Shifting proportions upon aging
B1 (♀)	Long/slim body and legs Decreasing incidence	Longer chest
B2 (♀)	Big upper body; short legs Incidence independent of age	Longer upper body
F1	Small girths; slim upper body Decreasing incidence	Longer upper body and shorter lower body
F2	Small arms; long chest Incidence independent of age	Shorter and broader legs
F3	Big upper body; big thighs Slightly increasing incidence	Shorter and leaner legs
F4	Massive body; big girths Incidence independent of age	Leaner legs
F5	Short upper body Increasing incidence	Longer chest
F6	Broad drooping shoulders Increasing incidence	Shorter and leaner legs; smaller arms and neck
B1 (♂)	Long/slim body and legs Decreasing incidence	(Longer chest)
B2 (♂)	Big upper body; short legs Increasing incidence	Leaner legs (and shorter upper body)
M1	Slim body; long extremities Decreasing incidence until 60 years	(Shorter upper body)
M2	Long upper body Incidence independent of age	Longer chest
M3	Big arms and neck Incidence independent of age	Shorter and leaner legs
M4	Broad neck; short legs Incidence independent of age	(Broader neck and upper body)
M5	Massive upper body Incidence independent of age	Longer chest; shorter and leaner lower body
M6	Long body; short extremities Incidence independent of age	Longer and bigger upper body
M7	Broad neck; thin legs Incidence independent of age	(Broader neck)

Men and women in mixed-gender body types (B1 and B2) are considered separately. Shifting proportions between younger (40–49 years) and older (70–80 years) participants of a body type are to be interpreted in relation to body height. Minor effects are given within brackets.

### The incidence of body types is a function of age

**Fig. 4:**
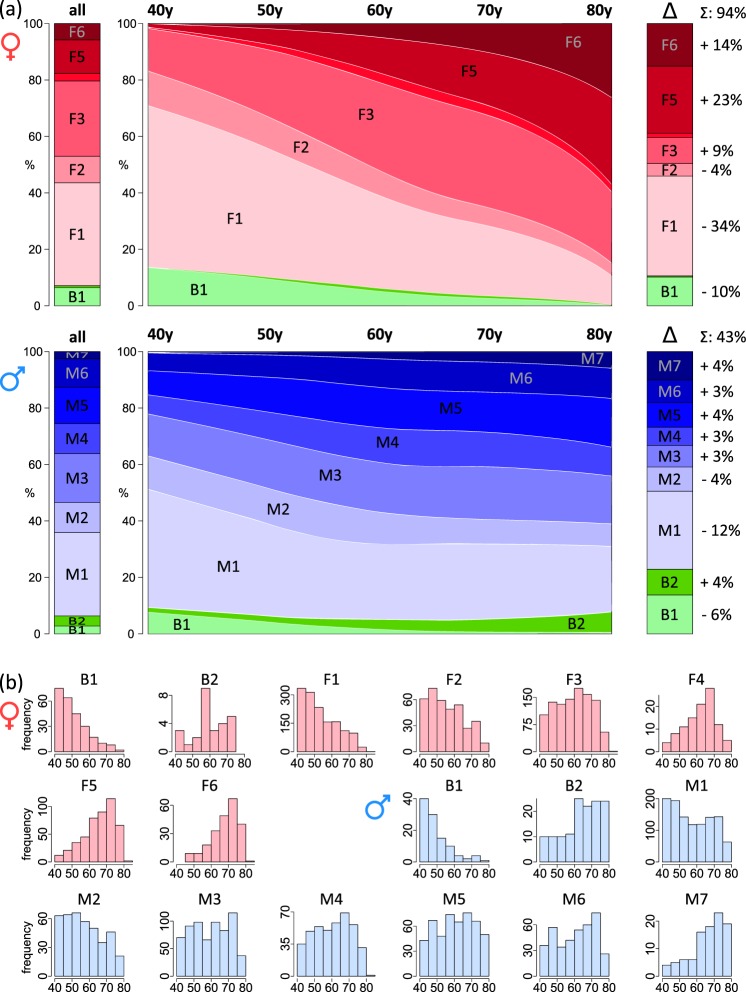
The distribution of body types reveals a systematic shift from young age to older age body types. **a** Percentage distribution of participants per body types as a function of age (middle part). The sidebars show the respective percentages of individuals in each of the body types averaged over all ages (left side) and their changes between the latest and earliest age interval (right side), respectively. **b** Frequency distribution of age of the participants collected in the individual body types.

The mean age of the body types ranges from about 45 to more than 65 years (Fig. 3a and Supplementary Fig. 6), reflecting a systematic change of the age distribution of the respective participants. The incidence of most of the body types markedly alters with age and locally deviates from the mean incidence, especially for younger and older people (Fig. 4a). Age-dependent changes are more pronounced for women than for men as indicated by the steeper slope of the respective curves in Fig. 4a. It corresponds to the stronger correlation of most of the meta-measures with age observed for women (Fig. 2b). Net changes of the relative frequency of body types are all together roughly twice as much in women (±94%) as in men (±43%; Fig. 4a, right part). Changes of the incidences of F-typess are observed in the complete age range, while the incidence of most M-types remains virtually constant above 60 years.

The body types B1, F1, and M1 show the highest incidence for middle-aged people of about 40–50 years, while their incidences then markedly decrease for elderly people, who enrich in F6 and M7. The incidence of F2, M2, and M3 is virtually independent of age. These body types collect participants from intermediate age ranges, which suggest compensation of in- and out-fluxes of type members upon aging.

Taken together, we find gender-specific aging of body shape where alterations of women are more pronounced than shape changes of men. Aging is characterized by the redistributions of body shapes towards specific body types of elderly people showing a narrower age distribution than body types of younger people (Fig. 4b).

### Transitions between the body types suggest Life Course trajectories

**Fig. 5:**
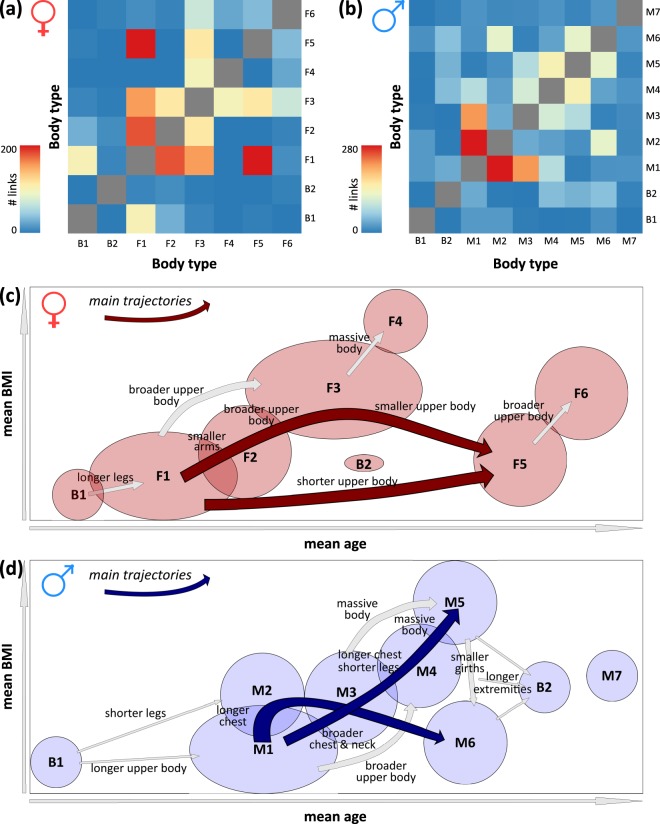
Similarity links suggest an age course of body types. Frequencies of similarity links between female (**a**) and male (**b**) body types are shown as heatmaps. These link frequencies were used to construct a schematic overview of transitions between the body types in an age-versus-BMI coordinate system. It suggests a partly linear sequence of female body types (**c**), and a more compact structure for male ones with more mutual transitions between the different body types (**d**). Intersecting areas and arrow widths approximately scale with the number of corresponding links (see also Supplementary Figs. 6 and 7 for more details). The figures schematically illustrate the mean BMI and mean age of the body types in an arbitrary scale.

So far, age-dependent alterations are described by changes of the mean meta-measures (Supplementary Fig. 6) and by the changing incidences of the body types (Fig. 4a). Both effects are linked because alterations of the meta-measures potentially change the incidences of the body types due to the re-classification of individuals between them. We applied a probabilistic approach to estimate such transitions. They are assumed to refer to pairs of individuals from two different body types with similar body shapes and they are obtained by counting all such similarity links between all pairwise combinations of body types (see heatmaps in Fig. 5a, b).

We found such links especially between “younger” body types (B1, M1, and M2 for men, and B1, F1, and F2 for women). Their number strongly decreases with increasing age (Supplementary text). Body types of the intermediate age range (M3–M5 and F3) form “transition” types linking “younger” with the “older” body types. The links typically refer to a relative shortening and broadening of the upper body (decreasing meta-measure H, increasing meta-measures B, K, and L; see Supplementary text). For example, the intermediate position of F3 suggests transitions from F1 and F2 towards F3 and from F3 towards F5 and F6 (see Fig. 5c). For men, links reflect a less pronounced age structure (Fig. 5d) in correspondence with the weaker age-dependent changes of M-types (Fig. 4a). Interestingly, F4 (women with massive bodies and thick girts) forms a relatively isolated body type virtually without similarity links to other body types. Younger women of the androgynous body type B1 link to F1, and younger men of B1 link to M1, whereby all of them collect slim bodies. The second androgynous body type B2 (big upper body) links to M4–M6, which all show larger upper body dimensions. In summary, similarity relations between individuals of different body types enabled us to identify possible transitions between the body types upon aging. They can be summarized into two major Life Course trajectories for women linking the younger and older body types F1 and F5, respectively, either by direct transitions or via the obese type F3, both affecting predominantly the dimensions of the upper body. For men, possible trajectories are more diverse, involve more inter-linked body types, and affect different parts of the body. Also for men one of this pattern of links reflects two major Life Courses, one via the obese type M5 and the other via the tall types M2 and M6.

### Body types diversify the aging curves of anthropometric indices

**Fig. 6:**
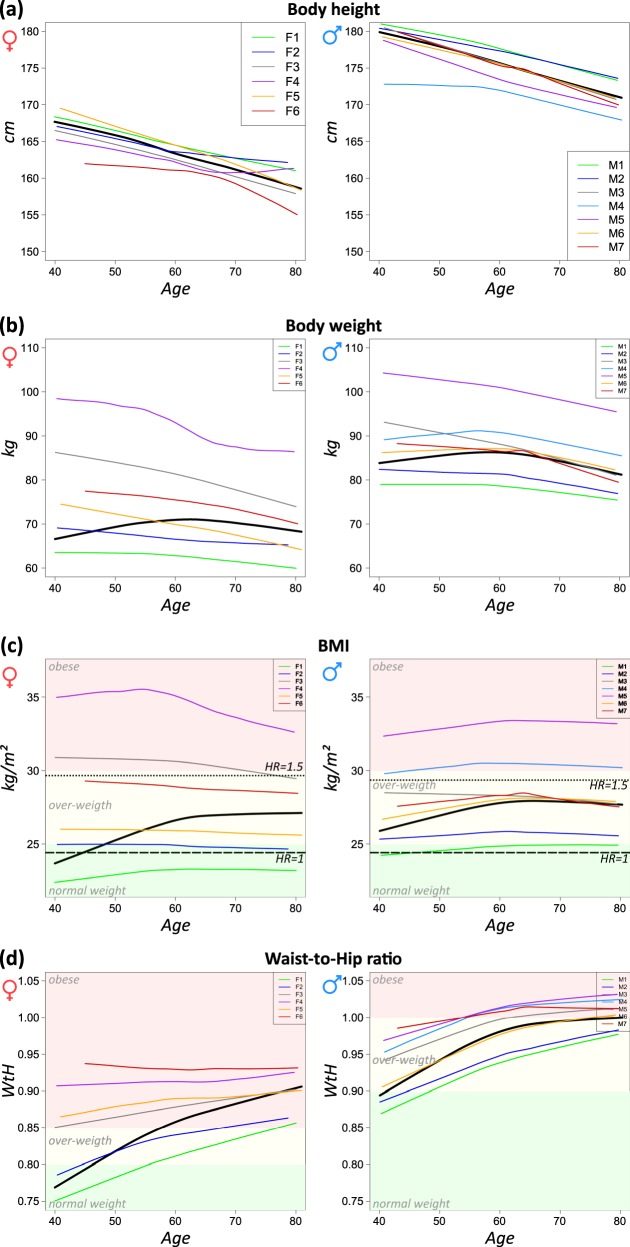
Body height, weight, BMI, and WTH of body types as a function of age. Curves were smoothed for each body type. Thick black lines indicate the mean parameter development averaged over all female (left panels) and male participants (right panels), respectively. Gender-unspecific body types B1 and B2 are not shown here due to low sample sizes. The background colors in **c**, **d** indicate different weight categories as indicated. The horizontal dashed line refers to minimum BMI-associated all-cause mortality (BMI = 24 kg/m^2^) and the dotted lines to hazard ratios of 1.5 taken from ref. ^25^.

We found that most body types develop specifically upon aging. For their better characterization, we decomposed the age dependencies of the “classical body indices” separately for each body type (Fig. 6). The body type-specific curves of body height roughly follow the course of the mean body height averaged over all participants (thick curve) with a slight scatter between them reflecting different body height levels. In contrast, the body weight and especially BMI curves of the individual body types show much stronger scattering (Fig. 6b, c).

Importantly, the BMI of the body types remains virtually constant, while the overall mean BMI increases until the age of 60–65 years. In other words, body typing roughly stratifies the population into virtually age-independent BMI levels, especially for women in the order F1 < F2 < F5 < F6 < F3 < F4 and, to a less degree for men M1 < M2 < (M3 ≈ M6 ≈ M7) < M4 < M5. M3, M6, and M7 are characterized by similar BMI levels (and body height), but different WTH levels. The relative small scatter between the body height curves of the individual body types indicates that body height is only a relatively weak determinant of body typing, while the much larger spread of the weight curves reflects its larger impact on BMI.

The WTH index is steadily increasing with age in most body types, reflecting a general apple-to-pear-like shift of body shapes, where the slope is largest for F-types with smallest WTH (F1, F2). WTH seems less suited as an age-independent marker index of body shape. The slope of the different WTH curves decreases with increasing WTH level, especially for women, leading to convergence of WTH indices and thus of stable pear-like body shape for elderly people. F6, accumulating elderly women, shows virtually constant WTH over age, and M5, accumulating obese men, is even slightly decreasing for participants older than 60 years. Interestingly, the female body type with the highest BMI, F3, does not show the largest WTH values, presumably reflecting a different fat distribution. Also, other body types of both sexes show differing relative BMI and WTH levels.

The mean BMI levels of F2 and M1 roughly correspond to a BMI value of about 24 kg/m^2^, which associates with minimum all-cause mortality^25^. The more obese types F4, M4, and especially F3 and M5, seem to associate with an increased risk based on previous data linking mortality risk and BMI^25^. Overall, stratification of body shapes into distinct body types levels out age-related alterations of body indices and enables the study of health-related associations in terms of defined anthropometric groups.

### Association between body types, physical activity, and selected health and lifestyle factors

**Fig. 7:**
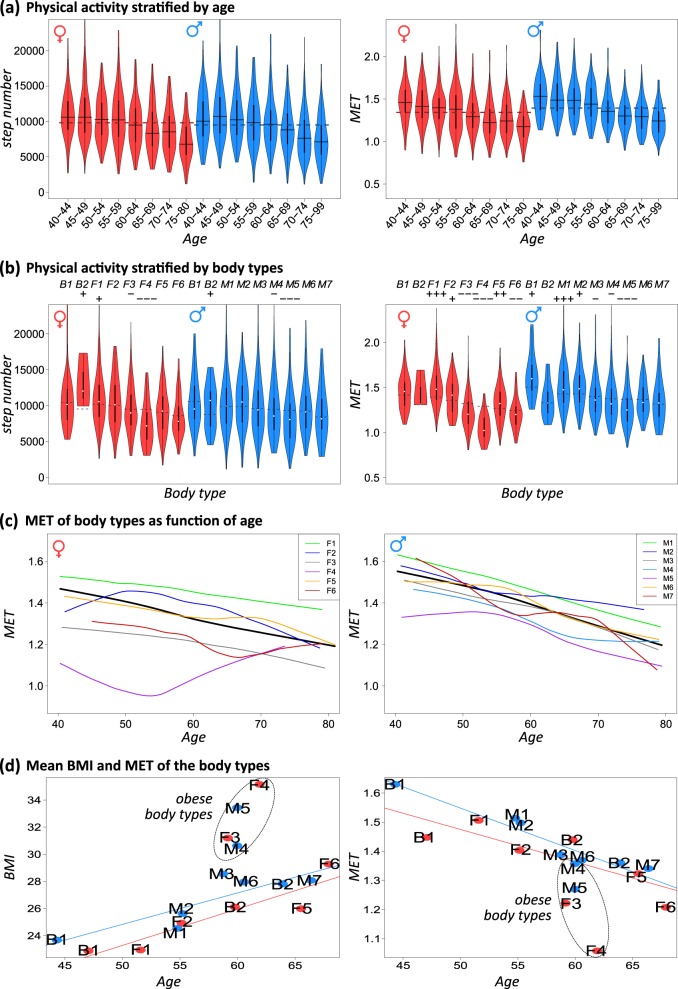
Physical activity of body types. **a** Physical activity as measured in units of the number of steps per day and MET decreases with age. **b** Body types divide into more and less active ones, where the former category collects younger and less obese individuals. Dashed lines indicate median values of the reference age groups, “+” and “−” symbols in the head line indicate significant differences between the body types and their reference groups with *p* values of <0.1 (+/−), <0.01 (++/–) and <0.001 (+++/−−−), respectively. **c** MET of the body types as a function of age resemble the respective BMI curves in Fig. 6 and reflects that high BMI associates with low physical activity. **d** The plot of body types’ mean BMI as a function of their mean age can be roughly described by lines of similar positive slopes for women and men (≈0.25 kg/m^2^ per year) if one excludes the obese types F3, F4, M5, and M4. MET provides negative slopes with larger variability of the values of the F-types and F4 as outlier showing lowest MET value.

Next, we studied the physical activity of the participants of the LIFE study as a function of age and its association to the body types. The number of steps per day and the metabolic equivalent (MET) as measures of physical activity systematically decrease with age similarly for women and men (Fig. 7a). Among the body types, we identified more (F1, M1) and less (F3, F4, M5) active ones using age-matched reference groups for comparison (*p* value <0.001, Wilcoxon’s rank-sum test, Fig. 7b). The mean MET value of the body types decreases as a function of age, except for the most obese type F4 (Fig. 7c). The MET levels anti-correlate with BMI and weight values (compare with Fig. 6, *r* = −0.87). The plots of the mean BMI and MET values per body type as a function of their mean age can be roughly described by lines of opposite slopes (Fig. 7d), but obese body types (F3, F4, M5 and to a less degree, M3, M4) deviate from these lines towards low MET, while B2F has a slightly elevated MET value. Note that MET is normalized per kg of body weight. Consequently, the treated energy grows not in parallel to body weight of the participants. Low MET values were found particularly for F3, F4, and also M5, which were risk groups in terms of high BMI (see above).

**Fig. 8:**
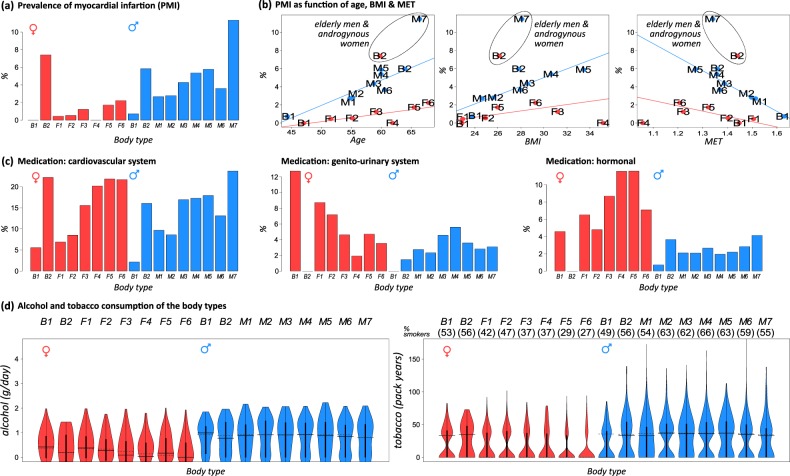
Myocardial infarction prevalence, lifestyle factors, and medication in body types. **a** Prevalence of myocardial infarction is much smaller for female (1.1%) compared with male (4.1%) participants of the LIFE study where however women of the B2F type have a strikingly high PMI value (7.4%). **b** Plots of PMI as a function of age, BMI, and MET consequently reveal much steeper slopes for M-types than for F-types. Women of the androgynous body type B2F are disproportionately affected by myocardial infarction. **c** Medication frequency of the individuals of the body types (within 7 days before their examination in LIFE) with drugs of the ATC groups C (cardiovascular system), G (genito-urinary system and sex hormones), and H (systemic hormonal preparations, excluding sex hormones and insulins), which all show anomalies for B2F. **d** Violin plots of the lifestyle factors alcohol consumption and smoking stratified by the body types. Alcohol consumption is higher for men than for women, and it slightly decreases with the mean age of the body types. Smoking among B-type women is more intense than among the other F-types and resembles that of men. Note that the violin plots reflect a bi-modal distribution for most of body types referring to non-smokers and smokers, respectively (“smokers” here subsumes current and former smoking; see percentage of smokers in the body type as indicated in the header).

We use the history of myocardial infarction (i.e., the prevalence of myocardial infarction in the previous life of participants, PMI) as one proxy to estimate the health risk of the body types. PMI of M-types markedly exceeds PMI in nearly all F-types, except for B2F (Fig. 8a). PMI steeply increases with the mean age for men’s types, but to a markedly less degree for women (Fig. 8b). We find slightly increased PMI for obese risk types of men (M5), while PMI is maximal for M7, presumably because of the increased mean age of this type (66.7 years). Age is obviously a relevant risk factor for elderly men compared to BMI and physical activity. Among women, the androgynous-type B2F shows strikingly high PMI. Notably, PMI is virtually independent of BMI and MET for women of all body types, except for B2F, while it increases/decreases with BMI/MET for men’s body types. Surprisingly, no case of previous myocardial infarction is among F4 collecting obese and elderly women (*p* = 0.11). Also, F3, another obese body type, associates with a relatively small PMI level. On the other hand, F3 and F4 collect, on the average, younger women than F5 and F6, suggesting that age constitutes the more relevant risk factor of PMI for women in contrast to men, who are under increased risk with increasing BMI for most body types.

The high PMI of the female B2F group is noteworthy, and it even exceeds the PMI levels of men’s body types (except M7). Androgynous women are obviously under elevated risk for myocardial infarction. To better understand this anomaly, we included alcohol consumption and smoking status as lifestyle factors, and also medication data available for the LIFE participants into our analysis. Particularly, medication of the group “C: cardiovascular system” according to ATC (Anatomical Therapeutic Chemical) Classification shows similar patterns as PMI, for example, higher percentage of medication in B2F and M7 (Fig. 8c). Obese and elderly women of body types F4–F6 take more medication than men of all BMI categories, except for oldest (M7) men. B2F women, on the other hand, take virtually no drugs of the medication categories “G: genito-urinary system and sex hormones” and “H: Systemic hormonal preparations, excluding sex hormones and insulins,” which considerably deviates from women of the other F-types. Women consume, on average, less alcohol than men, and the consumption decreases for body types of elderly women, but without marked specifics for B2F individuals (Fig. 8d). In contrast, B2F women show highest smoking level among women, which is comparable with that of men: 56% in B2F compared with 40% for all women and 59% for all men.

In summary, the physical activity of participants measured in units of MET anti-correlates with BMI and decays with age. Prevalence of myocardial infarction increases with age and/or BMI among men, but it is low among women, except those of the androgynous body type B2F, which, in turn, associates with high medication of group C drugs and relative extensive smoking.

## Discussion

Obesity per se is associated with health risk, for example, for cardiovascular diseases. This relationship is however complex due to several independent associations with risk factors^26–28^. Hence, the total body fat mass can have divergent associations. There is emerging understanding that instead of considering “simply” obesity, one hast to take into account “obesities,” that is, a heterogeneous multitude of phenotypes depending on factors such as the distribution of fat over different types of fat depots in the human body with different impact for different diseases and health in an age- and sex-dependent fashion^2^. “Paradoxically,” previous studies reported differences in short-, medium-, and long-term mortality showing partly better survival for overweight compared with normal and underweight individuals^29^. Moreover, despite perceptions of higher risk, increasingly obese patients can have fewer adverse clinical outcomes than expected^30^. Hence, there is a need for better risk assessment for obesity. This task includes collection of extended and refined cohort data to better understand variability between humans and also the identification of improved measures, which more specifically and sensitively serve as markers for health risks.

Single anthropometric indices, such as weight-to-height ratio, waist circumference, WTH, and BMI, are advantageous because they are easy to measure and to interpret in terms of simple threshold values^31,32^. As the major trend of “classical” anthropometry upon aging, we observed a decrease of body height, a moderate increase of BMI towards obese and overweight characteristics, and the increase of WTH ratio reflecting changes, popularly described as apple-to-pear-like body shape transformations. Application of 3D body scanning in combination with our body typing shows that human body shapes are diverse requiring a detailed description to study possible associations with health and lifestyle factors as a function of age.

Here we applied our body typing approach to resolve the multidimensionality of anthropometrical data extracted from 3D body scanner measurements. Our method reduces the about 150 derived body measures into 13 relevant anthropometric dimensions called “meta-measures.” These meta-measures reflect marked sex-specific differences resulting in the clear division into F (female) and M (male) body types. They were used to diversify human body shapes into 15 body types, 6 of them for female participants, 7 for male ones, and 2 mixed-gender types (see ref. ^18^ for details). Interestingly, aging results in virtually sex-independent reshaping of female and male bodies, which is characterized by the shortening and widening of upper body dimensions and an expansion of the leg girths of women and arm girths of men. With increasing age, slim body shapes remain slim, whereas obese body shapes tend to remain obese. We find marked sex differences of the distribution of body types upon aging: the populations of F-types change twice as large, in terms of cumulative percentages, compared with those of men. The age dependencies of the mean anthropometric indices (BMI, WTH) do not reveal such differences. Making use of similarities between the body measures of different body types, we deduced possible Life Course trajectories based on the frequencies of possible transitions between the types. For women and men, we identified two main aging paths referring to more obese and to normal weight individuals, respectively. For men, trajectories seem more complex particularly because of a network-like structure of links between the body types. Overall, female body shapes are more diverse and change more strongly than male ones. Anthropometric changes in terms of body types and indices begin to level off in the age range between 55 and 60 years as a physiological characteristic of the aging body of elderly people.

Several anthropometric measures, such as BMI and WTH, but also waist-to-height ratio^33^ and “a body shape index” (ABSI)^34^, have been developed to judge the health status in terms of obesity, mortality, and biological age^35^ using simple, “one-dimensional” measures. For example, discussion has developed around the so-called “obesity-mortality paradox” stating that mortality shows a U-shaped dependence on BMI with a minimum at about 25 kg/m^2 ^^25^. Other studies showed that confounding factors and/or other anthropometric measures remove the protecting effect of body fat on risk^36^. Beyond this, there is growing evidence that a certain level of, for example, BMI (or other anthropometric indices) can associate with different disease risks due to differences between “metabolic healthy” and “metabolic unhealthy” physiological states^37^, and also because a series of confounders, for example, age, sex, genetic factors, cardio-metabolic fitness, and pre-existing diseases. Our body typing offers a multidimensional metrics of anthropometry, which aims to fully exploit the data provided by 3D laser scanning. Importantly, our body types divide men and women into strata of virtually age-independent mean BMI levels ranging from underweight to obese characteristics. On the other hand, some body types represent different body shapes for similar BMI levels, for example, the slightly overweight types M3, M6, and M7 (see Table 2).

We here used participant-matched data on the prevalence for myocardial infarction and physical activity as example features to estimate the possible impact of body types on their health and aging behavior. Overall, physical activity (MET) is inversely related to BMI and decreases with age. Obese body types reveal markedly small MET values reflecting low activity levels of these individuals. Prevalence for myocardial infarction roughly increases with mean age and mean BMI for most of the body types with roughly twice the rates for men compared to women. However, obese and “inactive” body types are virtually inconspicuous regarding myocardial infarctions in the disease history of the respective men (M3) and especially women (F4). Interestingly, the obese (high weight and high BMI) female body type F4 shows a decreased WTH value compared with, for example, F6 (Fig. 6). These differences suggest a larger proportion of less dangerous “leg fat” in F4, which eventually is related to reduced health risk and possibly explains the effect observed. On the other hand, elderly men (M7) and androgynous women in B2F had a higher prevalence of myocardial infarction (11% and 7%, respectively) compared with the other F and M body types with possible impact for risk prediction. These results demonstrate that body typing increases resolution regarding health-related strata. However, extensive research, particularly longitudinal follow-up studies and larger sample sizes, are needed to extract detailed associations between body types and health, especially in obesity research to make this information usable in healthcare.

We studied the aging body shape using 3D laser scanning data of about 8500 adult people of Middle European ethnicity randomly selected from the population of Leipzig, Germany. Ethnic differences in body size and composition have been identified as a limitation to use simple body indices for estimating health risk^38^. Systematically varying distributions of visceral and subcutaneous fat between populations are thought to associate with differences in their obesity status and health risks, for example, for developing cardio-metabolic diseases. It has been reported that Inuit and Africans have less fat for any given anthropometric measure compared with Europeans^39^. Hence, the relationship between conventional obesity measures, fat accumulation, body shape, and health risk is not clearly established across ethnic groups at present. In consequence, the use of conventional anthropometric measures in clinical practice to identify health risk is ethnicity dependent and therefore requires further research. Systematic body typing of so far understudied, non-middle European populations offers an option for the better, in-depth understanding of phenotypic variability of humans, its aging behavior, and its relation to health. We expect that the frequency distribution among our body types and their health risk differs between populations. One interesting question is whether our collection of body types must be extended by novel types in other ethnicities. 3D laser scanning offers a relatively simple and non-expansive method for such studies in future.

For estimating the “internal” fat distribution, computational tomography seems to be the best, but also the most expansive method, especially with its ability to pin-point differences between visceral and subcutaneous fat in the abdominal region^40^. DXA is another special x-ray-based method enabling to measure regional body composition with high precision and stability and showing advantages over classical, single-index anthropometry in estimating health risks^41,42^. Although the expose of patients and operators to ionizing radiation does not exceed natural background radiation, it usually requires licensing of the technologist and a designated X-ray site in contrast to optical laser scanning techniques. Despite such direct “competitive” pro-versus-contra comparisons, both DXA and laser scanning body typing provide complementary information about, for example, the “internal” fat distribution and the “external” body shape, respectively. Hence, parallel studies using both methods are required to achieve a better understanding of the physiological state of the body types, for example, by relating the body shape to the fat distribution and to the associated health risks. Interestingly, recent developments of DXA data analysis aim at deriving “holistic” body shapes from the measured “tissue distribution” in order to better describe its relation to health risk^43^. Hence, both 3D laser scanning-based anthropometry and DXA tend to make use of “body shape” concepts with “tissue distribution” and “body surface” information as outputs, respectively.

In summary, future directions can be seen in the systematic use of the concept of body types to describe body shapes, its application to different ethnicities to better describe the variability of human body shapes, combination of body shape measurements using different techniques to relate the external body shape to the “internal” tissue distribution, and the associations with health and aging.

Body typing by utilizing 3D body scanning data offers opportunities for studying the diversity of human phenotypes. Stratification of aging human body shapes into Life Courses is one step towards personalized anthropometry with the perspective to predict lifetime risks. Verification and further development of this approach should include longitudinal follow-up programs, wider phenotype association profiles to identify multidimensional anthropometrical risk profiles for clinics, and, last but not least, improved bioinformatics for dimension reduction and focused feature selection of this data type. High-dimensional anthropometry after further reduction of size and price of available scanning devices potentially provides a standard option in epidemiological research and, possibly, also in clinical practice for monitoring the health status of patients.

## Methods

### The LIFE study and ethics approval

Our analysis included data collected in the frame of the LIFE-ADULT study between 2011 and 2014 with a targeted sample size of 10,000 participants with uniform sex and age distribution in the age range between 40 and 80 years^17^. Lists of randomly sampled citizens along with officially registered age and sex were provided for recruitment by the resident’s registration office of the city of Leipzig. The study included persons with sufficient knowledge of German to read and understand the study documents and questionnaires. An existing pregnancy was exclusion criterion. All participants gave their written consent to participate in the study.

We utilized body scanner data of 8499 participants and physical activity measurements of 2429 participants. The study was approved by the responsible institutional ethics board of the Medical Faculty of the University of Leipzig.

### 3D body scanning and anthropometric data

3D body surface scanning was performed by a “Vitus Smart XXL” 3D laser scanner (Avalution GmbH, Kaiserslautern, Germany), which provides an image of the body surface of each participant. In total, 155 body measures were extracted from each of these images using AnthroScan 2.9.9 software in agreement with ISO 20685, the international anthropometric database standard for 3D scanning methodologies.

We considered 134 body measures of 8499 participants, including length and girth measures, weight, and the indices “BMI”^44^, “WtH”^24^, WHtR^33^, and “ABSI”^34^. The body measures of each participant were divided by the body height to get height-normalized values. Each measure was then *Z*-normalized, which makes the different measures comparable. Details about data preprocessing are given in ref. ^18^.

### Meta-measures and body types

We analyzed 3D body scanning data based on self-organizing map machine learning to stratify the LIFE-ADULT data into body types^18^. In brief, machine learning aggregates the body measures provided by the scanner software into a set of 13 meta-measures instead of 150 features measured by the scanner. Each meta-measure represents a cluster of correlated single-body measures. They define the relevant dimension of the body shape (see Table 3 and Fig. 2a for illustration). Approximately half of the meta-measures collects length measures (*n* = 7), while the other half mostly refers to girth measures (*n* = 5) of different parts of the body.

**Table 3:** List of meta-measures and most prominent associated body measures.

Meta-measure	Associated body measures
A. Shoulder width	Shoulder width, width of armpits, cross shoulder length
B. Upper body girths	Chest girth, waist girth, hip girth, belly circumference, BMI, WHtR
C. Thigh girth	Thigh girth (left and right)
D. Head circumference	Head circumference, crotch length
E. Shoulder angle	Shoulder angle (left and right)
F. Sideseam length	Sideseam length (left and right), ankle height (sideseam left and right), head height
G. Inseam length	Inseam length (left and right), ankle height (inseam left and right), crotch height
H. Upper body lengths	Distance neck to hip, distance neck to knee, distance waist to knee
I. Arm length	Arm length (left and right), up arm length (left and right)
J. Neck length	Neck length
K. Neck girth	Neck girth (at base and middle)
L. Arm girth	Arm girth (forearm, up arm, elbow; each left, and right), ankle girths (left and right)
M. Torso length	Neck to waist distances (left, right, central)

Meta-measures are labeled with capital letters.

The meta-measures were then used to cluster the participants of the study into 15 body types (see Supplementary Fig. 6a, c for illustration): two of the body types (B1 and B2) lack gender specifics because they include both male and female participants. Six body types (F1–F6) collect almost exclusively women (F1–F6), while seven body types (M1–M7) are male specific. The body types differ in the mean age, body height, weight, and BMI characteristics of the participants^18^. We defined age-matched and sex-specific reference groups for each of the body types collecting all participants in an age window of ten years independent of their body type. Body types’ features, for example, BMI and activity parameters, were then compared with those of the respective reference group and tested for significance using Wilcoxon’s rank-sum test.

### Similarity links between body types

For comparison of the body types, we estimated their mutual similarities by calculating the Euclidian distance of the meta-measures between all pairwise combinations of participants. The number of most similar pairs then defines links between the corresponding body types. In Supplementary Material, we stratify body type links by age, and associate differential meta-measures to them (Supplementary Figs. 6 and 7). Changes of the body type with age are assumed to occur along these links.

### Physical activity data and MET estimation

Physical activity status of a subcohort of 2429 participants (1319 men and 1100 women) was estimated in units of MET using the SenseWear Pro Armband (SWA, Bodymedia Inc., Pittsburgh), a multi-sensor tool with 2-axis accelerometer, heat flux sensor, galvanic skin response sensor, skin temperature sensor, a near-body ambient temperature sensor, and heart rate detection using a chest strap^45^. It was used by participants on 8 days or more, including at least 4 weekdays and 1 weekend day^46^. We only consider days with sufficient wearing time of at least 18 h on weekdays, or at least 20 h on weekend days^47^. Under these conditions, the SWA delivers valid and reliable data as proven by several validation studies^46,48–50^. The SWA software estimates physical activity in units of MET, where a MET value of unity refers to the amount of oxygen consumed while sitting at total rest. It is set equal to 3.5 ml O_2_ per kg body weight and minute^51^. In addition to MET, we also used the “number of steps per day” of the participants counted by SWA during the measurement as a rough direct estimate of physical activity. The SWA has limitations leading to lower accuracy, for example, while cycling, and it cannot be worn while swimming^47^.

### History of myocardial infarction, medication, and lifestyle factors

PMI of LIFE participants was assessed in questionnaires and refers to at least one previous infarction during their lifetime according to self-reporting. These particular reports were further verified in an interview lead by a physician, who asked about details of the previous diagnosis of myocardial infarction. This “self-reported” PMI amounts to 4.3% of men and 1.2% of women in the LIFE-ADULT cohort. These percentages are lower than the mean lifetime prevalence for myocardial infarction of the German population (age range: 40–79 years) of 7% and 2.5%, respectively^52^. We are aware of possible inaccuracies in self-reporting giving rise to a bias of the PMI values used and possibly explains the deviation between the PMI in LIFE compared with that in the German population.

Further, we make use of selected lifestyle characteristics of the participants such as cigarette and alcohol consumption, and medication status collected via questionnaires.

## Supplementary information

Supplementary text

Report

## Data Availability

Participant data presented in this publication are not publicly available due to limitations of informed consent. However, these data are available from the corresponding author on reasonable request. The anthropometric data are available from “The Leipzig Health Atlas” repository under accession number 7XUCQW5V05-3.

## Code availability

Data analysis was performed using R 3.5.1 and the packages “vioplot” for violin plots, “fmsb” for polar diagrams, and standard package “stats” for Wilcoxon’s rank-sum test, regression models, and Loess curve smoothing.
