# Supplementary material for: The aging human body shape
Source: NPJ Aging Mech Dis. 2020 Mar 24;6:5. doi: 10.1038/s41514-020-0043-9 (PMC7093543; doi:10.1038/s41514-020-0043-9)

Ageing human body shapes

Alexander Frenzel, Markus Loeffler, Nadja Walter, Kerstin Wirkner, Hans Binder, Henry Loeffler-Wirth

Supplementary material

1.1. Characteristics of body types 2
1.2. BMI distributions of selected body types 3
1.3. Relation of selected body measures to MET 4
1.4. Meta-measures as a function of age 5
1.5. Ageing body types 7
1.6. Age-resolved similarity links between body types 9

1.1. Characteristics of body types

Supplementary Figure 1: (a) Surface images of individuals selected from each body type (see also [1]). (b) Fraction of participants older than 70 years in the body types. Depletion (- signs) and enrichment (+ signs) are estimated using Fishers exact test (p-values of <0.1 (+/-), <0.01 (++/--) and <0.001 (+++/---)). The fraction of elderly people is highest in F5 and F6 among women, in and M7 and B2 among men. Note that also the F-types collect a small number of men and the M-types a small number of women. These ‚cross-gender' assignments were not considered in the downstream analyses.

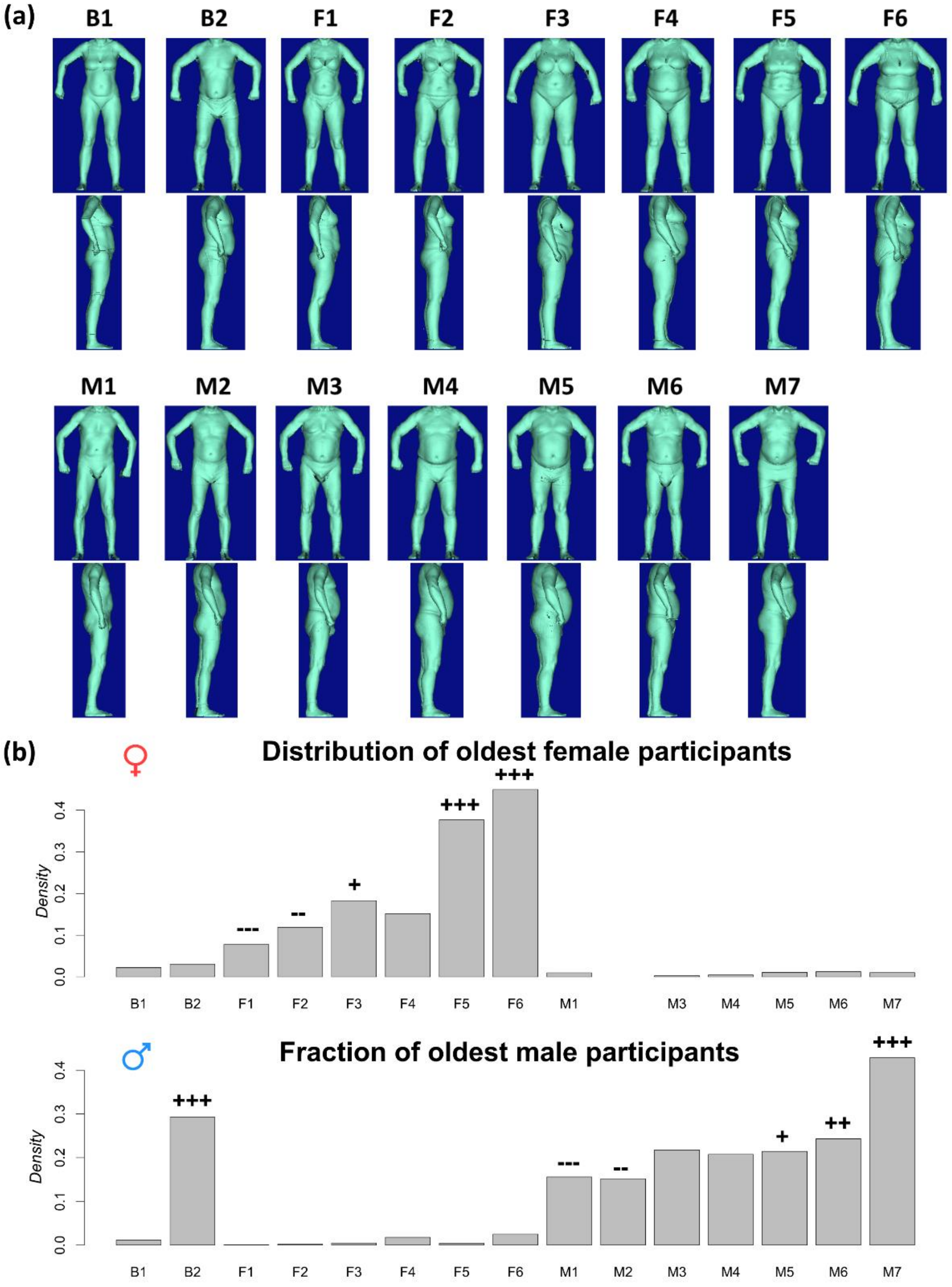

1.2. BMI distributions of selected body types

Supplementary Figure 2: Density distributions of selected body types over the BMI. F3 and M5 are characterized by widest distributions, while F2 and M7 with narrower distributions were shown for comparison. Note that the alterations of the mean BMI values of the body types are rather small compared with the variability of the distributions (see arrows).

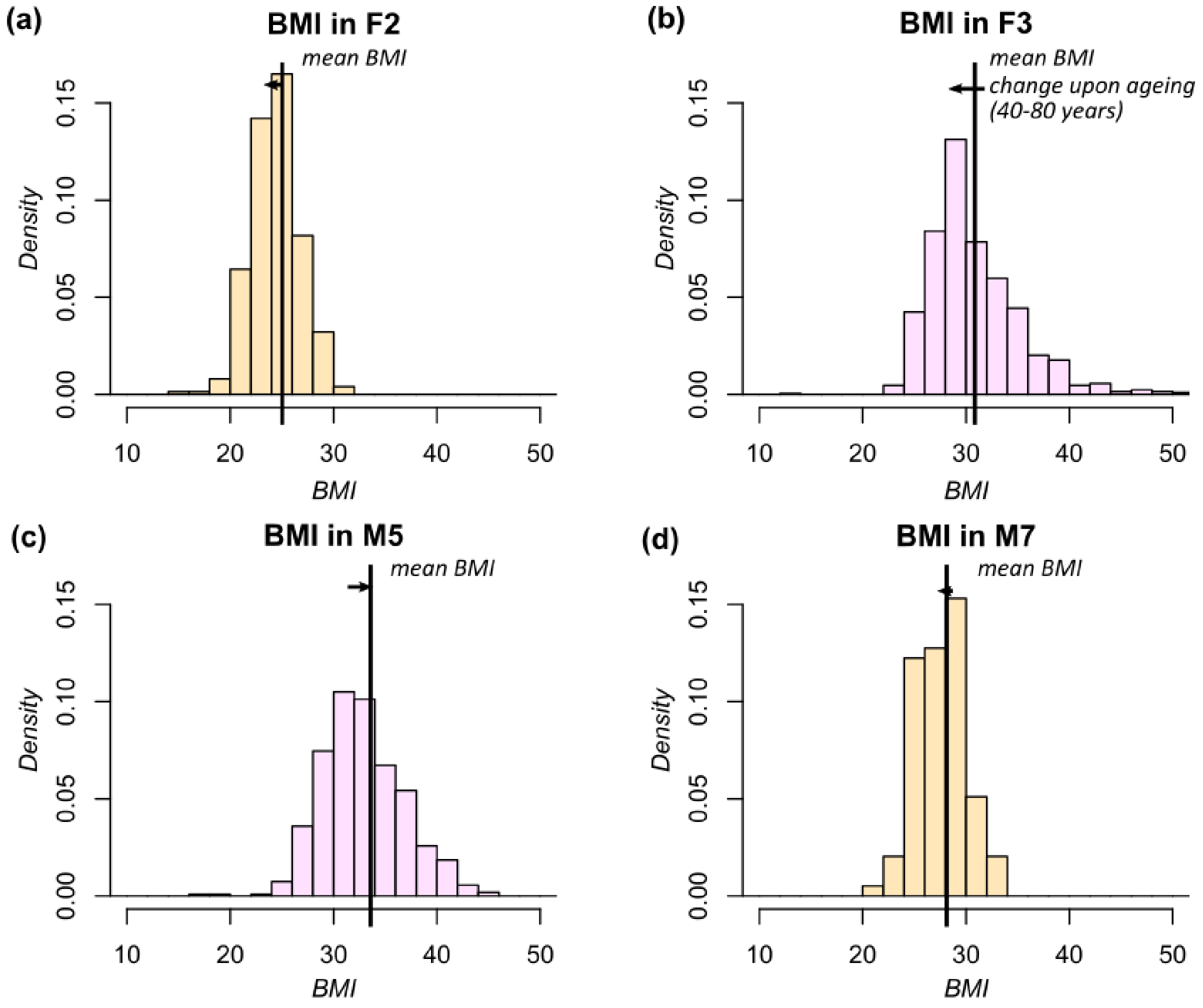

1.3. Relation of selected body measures to MET

Supplementary Figure 3: Bar plots represent values of body measures for less and more active participants (MET <=1.4 and >1.4, respectively). R^2 is given for linear models over all participants. We selected body measures of highest R^2. Accordingly, girth measures (including belly circumference) show strongest associations with physical activity while length measures (body height and arm length) virtually don‘t correlate with MET.

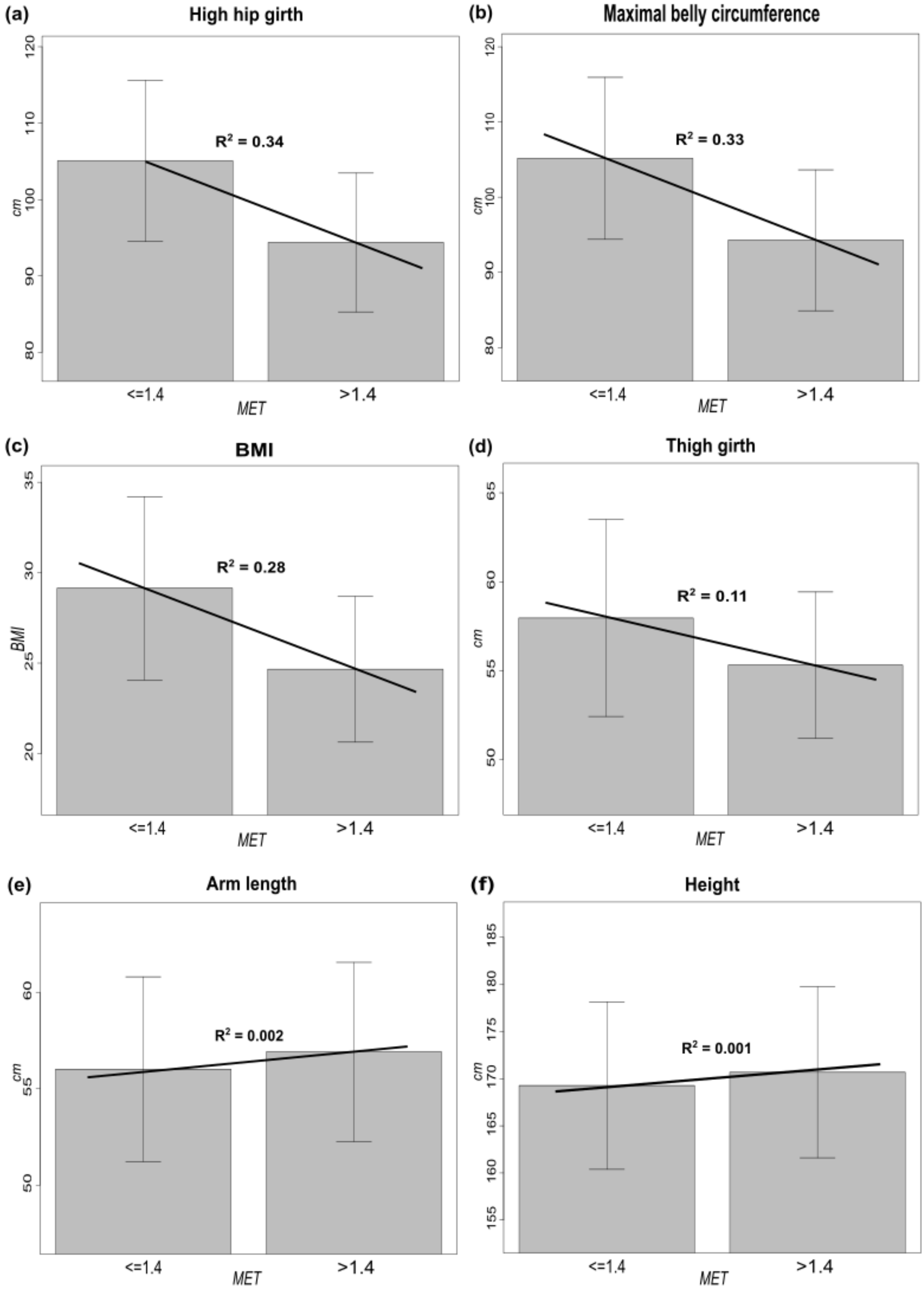

1.4. Meta-measures as a function of age

Supplementary Figure 4: Meta-measures are given as violin plots stratified by sex and age. Dashed horizontal lines refer to mean values in women and men, respectively.

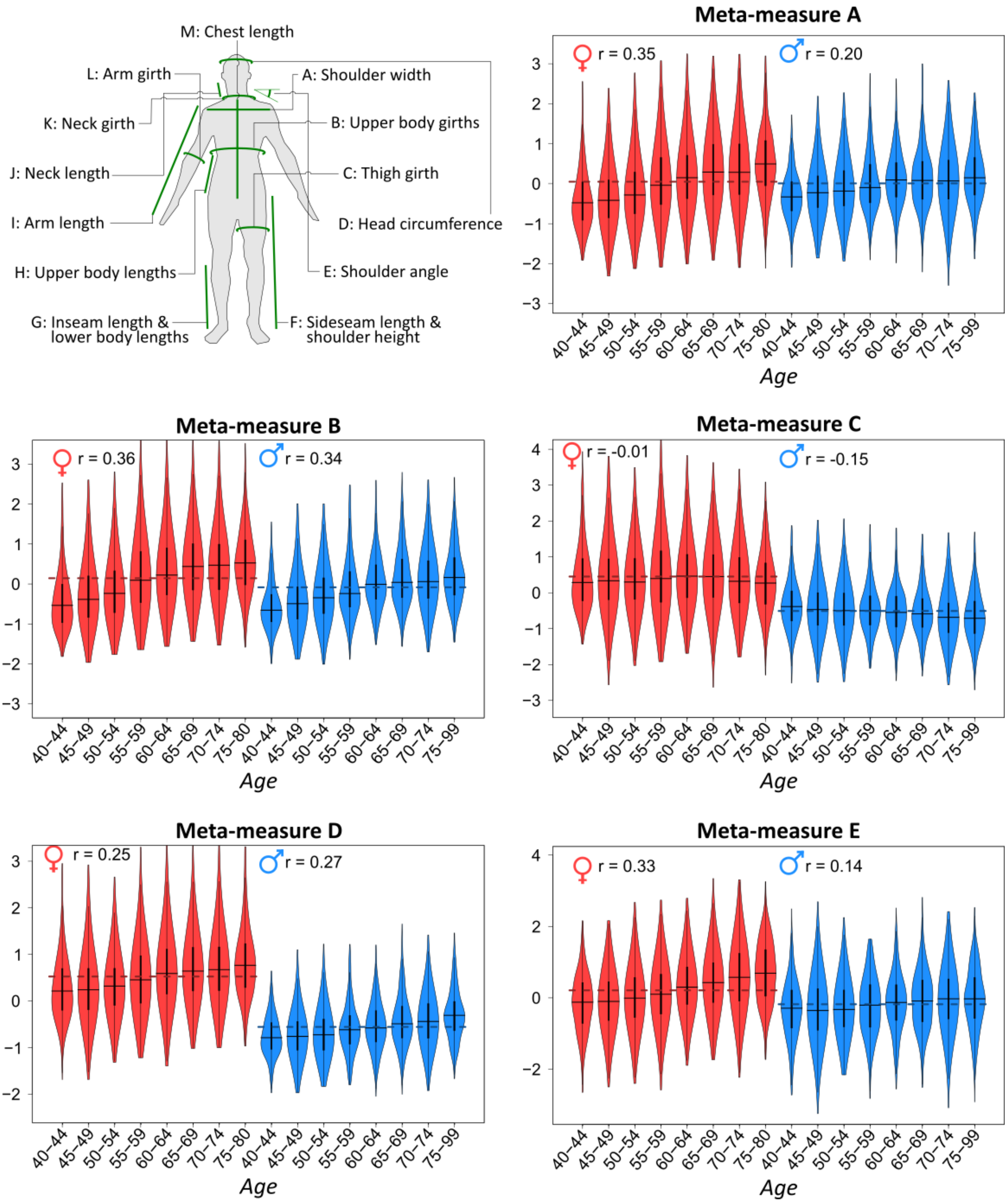

Supplementary Figure 5: Supplementary Figure 4 continued.

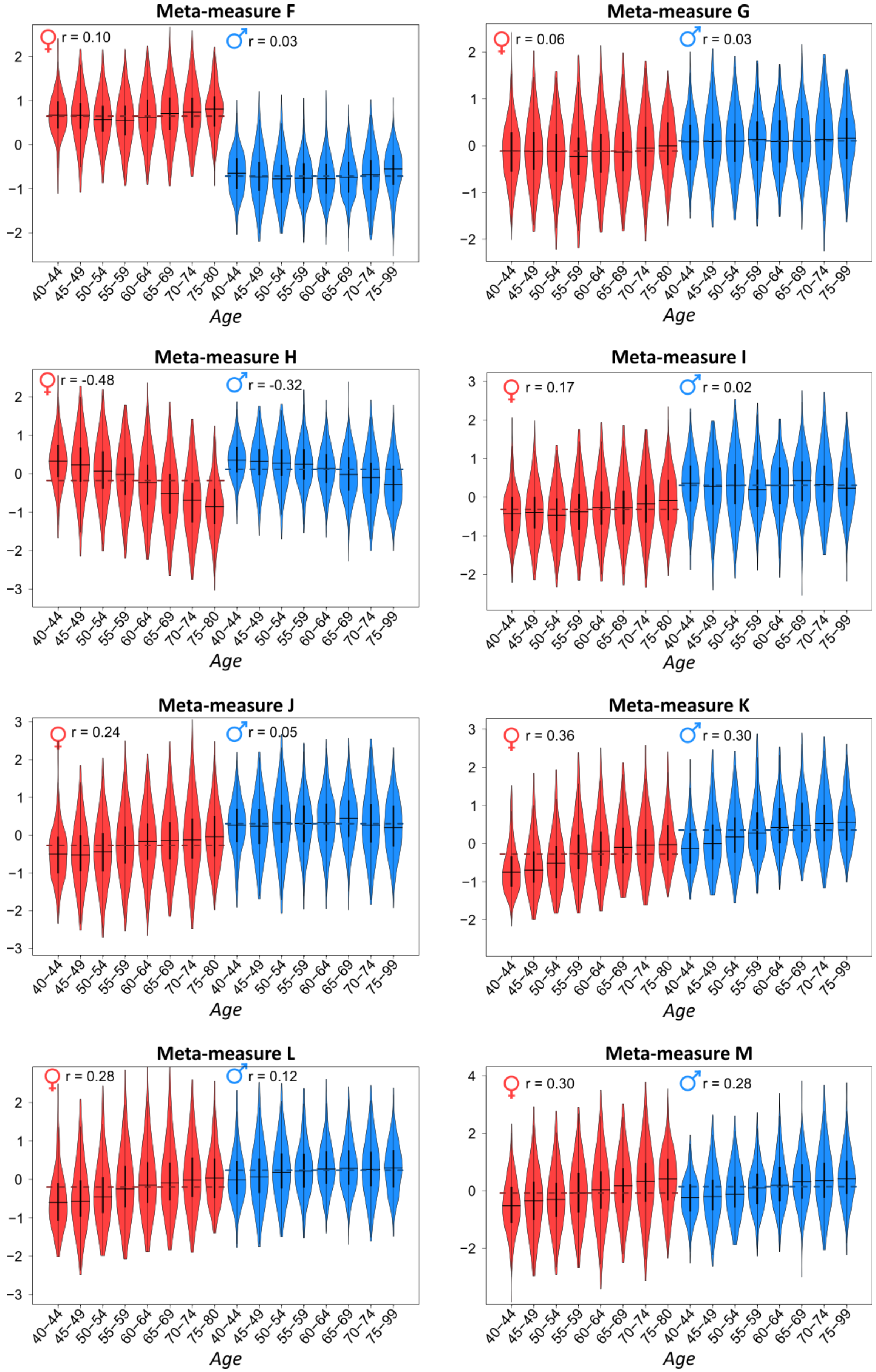

1.5. **Ageing body types**

Body grams of the body types reveal type-specific changes upon ageing (Supplementary Figure 6): Part of them are characterized mainly by increasing meta-measures (e.g. B2F, F5 & M6), while others are dominated by decreasing meta-measures (F3, F6 & M3) or show virtually age-invariant meta-measures (F2, M1, M4 & M7). In general, female body types seem to underlie stronger changes than male ones. The meta-measures can be divided into growing, shrinking and virtually invariant ones: The shoulder angle (E), for example, increases with age meaning more hanging shoulders for elderly people. Also, chest (I) and arm lengths (M) are growing measures reflecting the increase of the upper body. Decrease of the dimensions of the lower part of the body is reflected by decreasing thigh girths in men (C). Overall, ageing body types are characterized by the shift of body proportions towards a larger upper part and smaller legs, which become relatively short and lean.

Some of the meta-measures reveal gender-specific alterations, such as meta-measure B (upper body girths) which increases typically in the male body types this way reflecting the shift into apple-like body shapes. Other meta-measures, e.g., arm length (I), arm girths (J), neck girth (K) and neck length (L) specifically change in female body types and partly reflect the increase of the upper body's size. Note that B2F has a WTH value resembling more that of men than that of women (see Figure 3d in main document). The decrease of arm girths and neck length (J and L) in F6 indicates decreasing upper body proportions. The body types F6 and M7 collect the oldest participants with mean ages of 67.8 and 66.4 years, respectively (see Figure 3a in main document).

Supplementary Figure 6: Ageing body types: (a) and (d) show the number of participants, their mean age and weight per body type as defined in [10]. (b) and (e) show body type-specific bodygrams averaged over 10-years intervals given as overlays (see legend). Meta-measures with marked increase or decrease ($\Delta Z > \pm 0.5$) are highlighted by arrows. (c) and (f) show difference Δ-bodygrams visualize to the changes of the meta-measures between the 40-49 and 70-80 intervals of each body type.

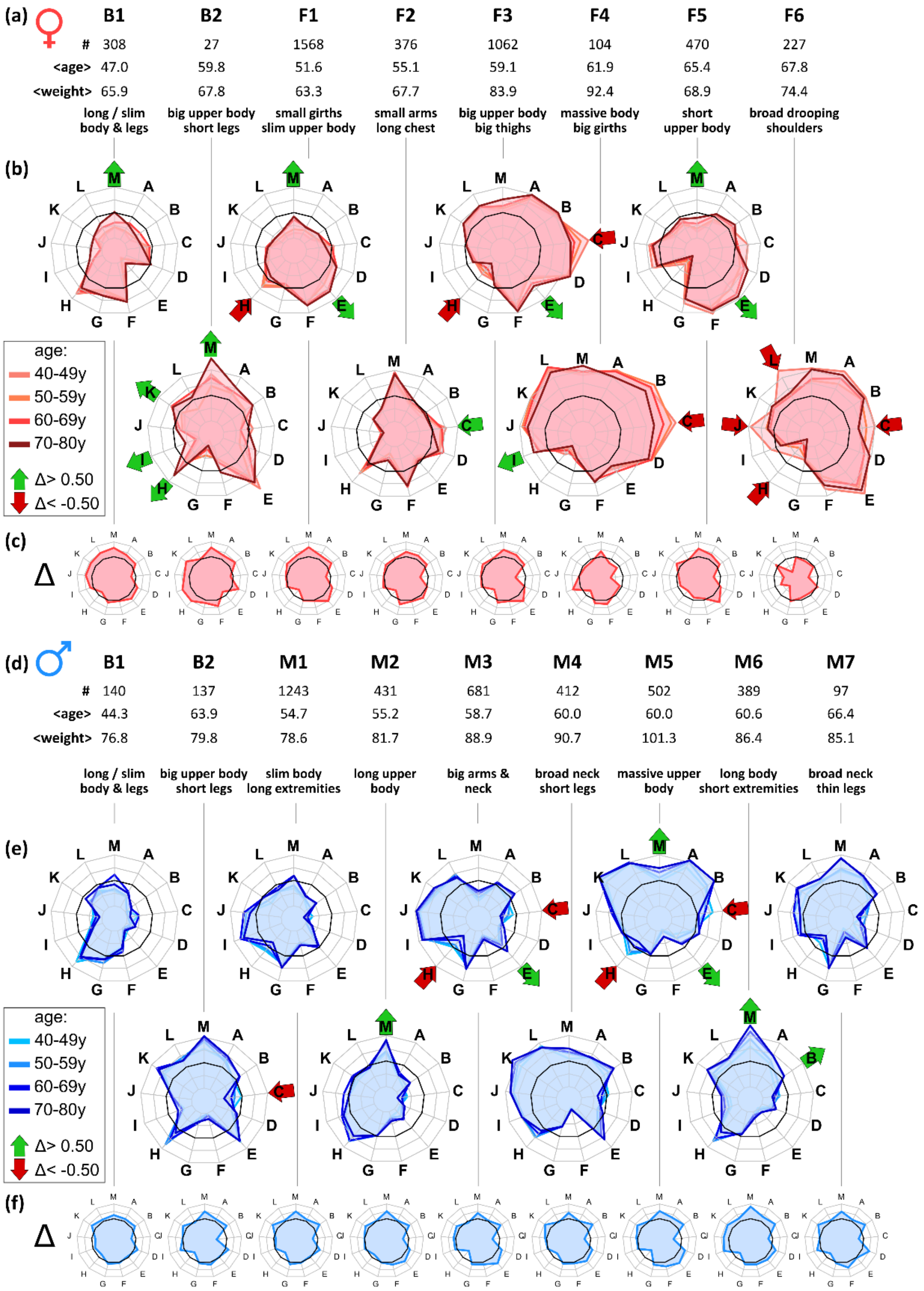

1.6. Age-resolved similarity links between body types

Similarity links are embodied by pairs of most-similar participants assigned to different body types. Further analysis was then restricted to frequent links with p-value < 0.05 (Fisher's exact test). Links between body types were associated with differential meta-measures showing consistent changes between the participants of the linked body types (p-value < 0.05, Wilcoxon rank-sum test).

We stratified the similarity links between all pairwise combinations of body types in 10 years intervals and visualized these links (Supplementary Figure 3).

Supplementary Figure 7: Connections between female body types are given, stratified by decadal intervals and separately for each body type. The respective reference body type is highlighted in each panel. Significantly differential meta-measures are assigned to majority of connections, indicating changes in body shape compared to the reference body type.

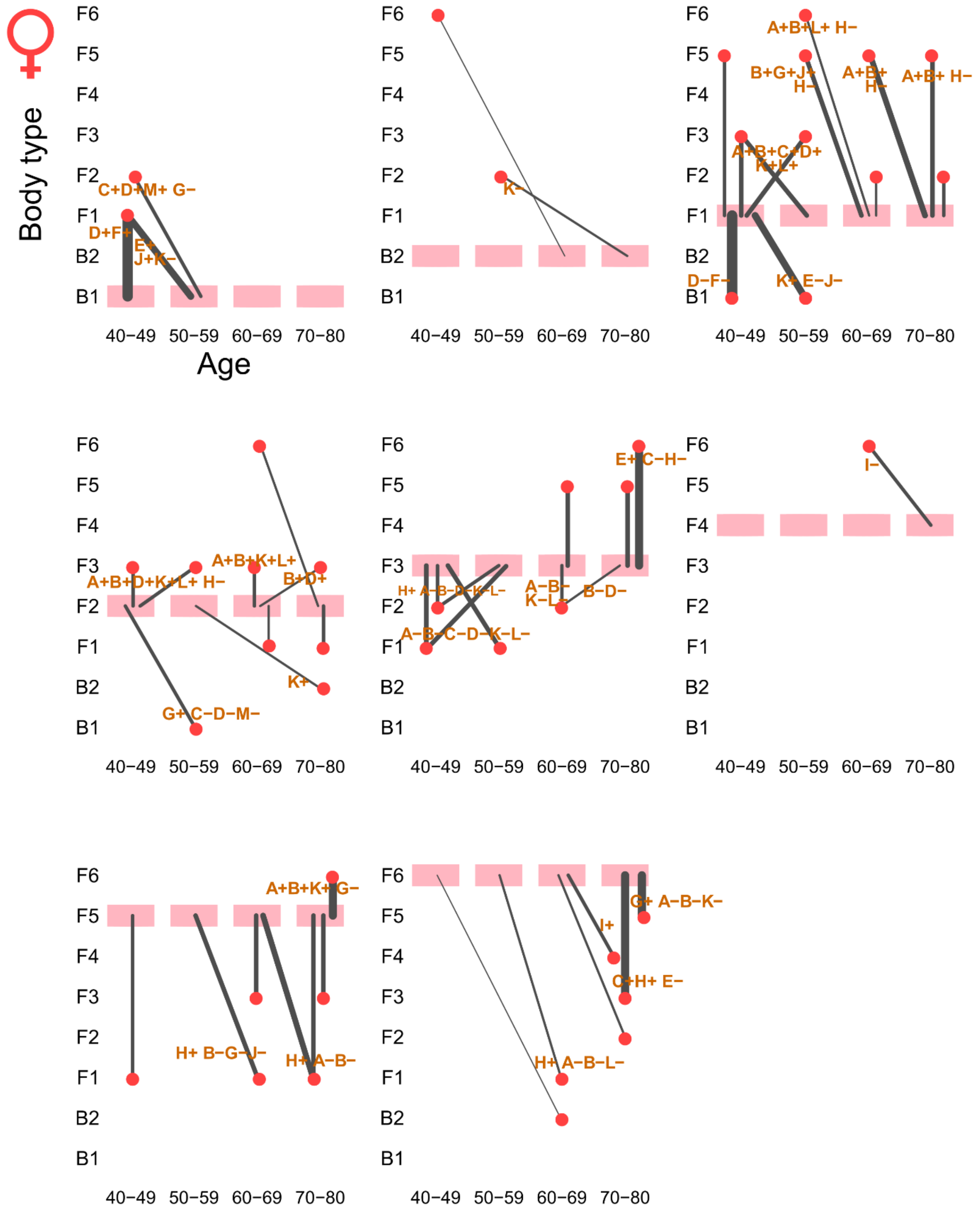

Supplementary Figure 8: Connections between male body types. See description of Supplementary Figure 7.

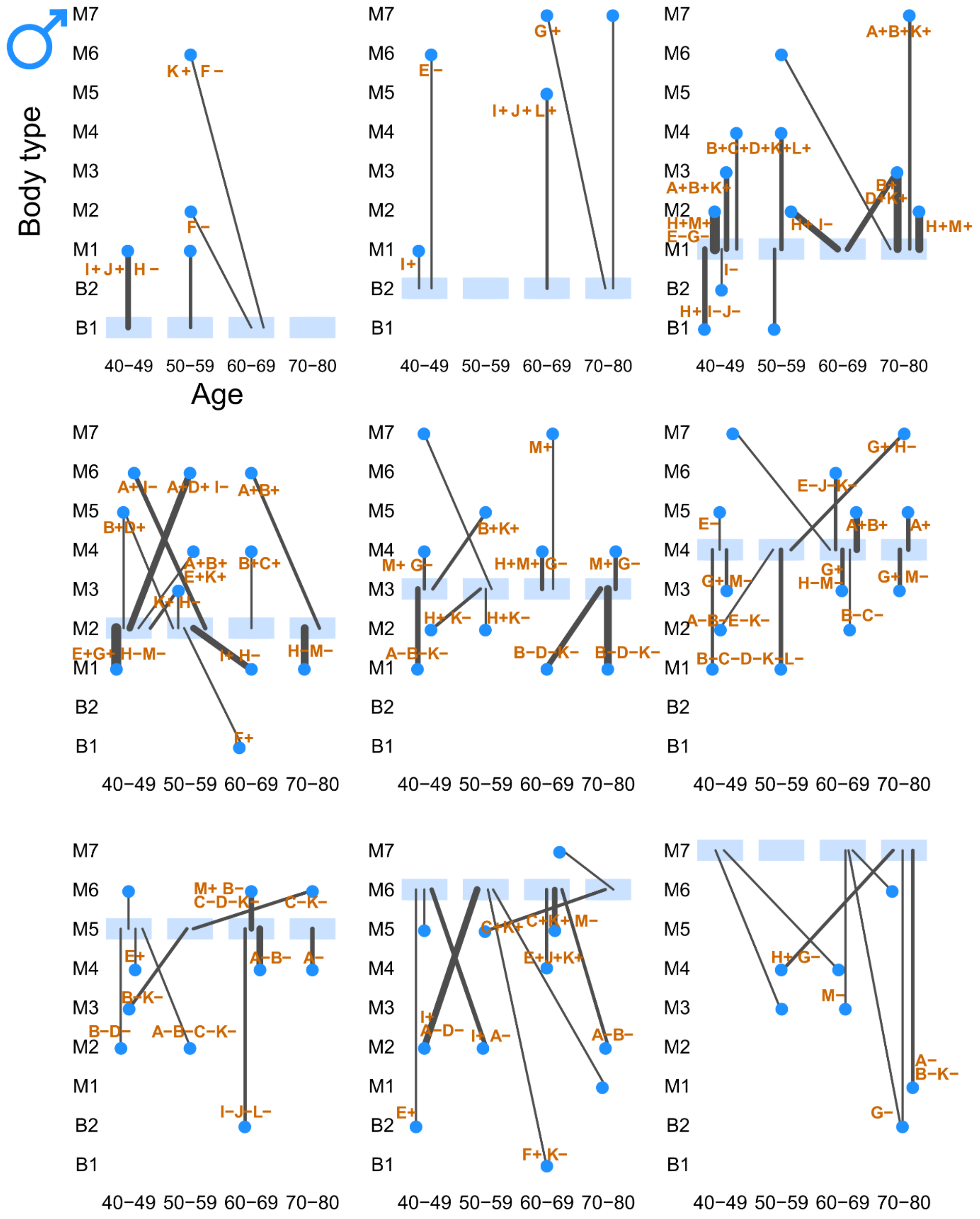

Supplement: Supplementary file 1 — Supplementary text [file 41514_2020_43_MOESM1_ESM.pdf]
